# Probe signal correction for differential methylation hybridization experiments

**DOI:** 10.1186/1471-2105-9-453

**Published:** 2008-10-23

**Authors:** Dustin P Potter, Pearlly Yan, Tim HM Huang, Shili Lin

**Affiliations:** 1 Human Cancer Genetics Program, OSU Comprehensive Cancer Center, The Ohio State University, Columbus, OH USA; 2 Department of Molecular Virology, Immunology, and Medical Genetics, The Ohio State University, 460 W. 12th Avenue, Columbus, OH USA; 3 Mathematical Biosciences Institute, The Ohio State University, 231 W. 18th Avenue, Columbus, OH USA; 4 Department of Statistics, The Ohio State University, 1598 Neil Avenue, Columbus, OH USA

## Abstract

**Background:**

Non-biological signal (or noise) has been the bane of microarray analysis. Hybridization effects related to probe-sequence composition and DNA dye-probe interactions have been observed in differential methylation hybridization (DMH) microarray experiments as well as other effects inherent to the DMH protocol.

**Results:**

We suggest two models to correct for non-biologically relevant probe signal with an overarching focus on probe-sequence composition. The estimated effects are evaluated and the strengths of the models are considered in the context of DMH analyses.

**Conclusion:**

The majority of estimated parameters were statistically significant in all considered models. Model selection for signal correction is based on interpretation of the estimated values and their biological significance.

## Background

With the advent of microarray technology, whole genome DNA methylation profiling has become a common approach to understand the systemic effects of this aberrant epigenomic mark in basic, translational, and clinical research. DNA methylation in vertebrates is a heritable somatic modification in which a methyl group is added to the cytosine residue of a CG dinucleotide. Significant accumulation of DNA methylation in critical regions of the genome correlates with respect to reduction in gene transcription. The human genome contains regions with higher than expected occurrence of CG dinucleotides which are called CpG islands or CGIs. Under normal conditions, the CGIs in the repeat regions are highly methylated whereas those found close to active gene promoters are free of methylation. This scenario reverses in diseased states (i.e., gain of methylation in single copy gene promoters and loss of methylation in repeat regions). In cancer samples, for example, aberrant DNA methylation occurs in the promoter region of tumor suppressor genes thereby contributing to cancer development and tumorogenisis [1,2]. As an explanation, it has been proposed that DNA methylation cooperates, both structurally and functionally, with chromatin modification in the repression of gene expression [3-5].

Two-color microarrays quantify the relative abundance of RNA or DNA between experimental samples. Recently microarrays have been employed more frequently to assay methylation profiles. The pixel intensity of the two colors can be interpreted as the amount of material hybridized to a given probe sequence. DNA arrays have been developed to interrogate the methylation signatures of the entire genome or at least focused regions such as CGIs. Two general experimental protocols have been developed to take advantage of these assays: methyl-DNA immunoprecipitation (meDIP) and differential methylation hybridization (DMH).

The meDIP methodology [6,7] employs antibodies specific for 5-methyl-cytosine residues to enrich methylated DNA fragments in the sample. The pull down DNA fragments are PCR-amplified and co-hybridized with a whole genome sample to generate a two-color image. This method has been successfully used by different groups; however, the antibody recognition motif is not well-defined thereby potentially biasing the experimental outcomes.

The DMH protocol [8,9] employs methylation-sensitive restriction enzymes as opposed to antibodies to investigate the methylation status of the genome. Sonicated DNA fragments are ligated to linkers and subsequently interrogated by these enzymes which will cleave any fragments containing unmethylated enzyme recognition sequence. The unrestricted fragments are PCR-amplified to generate a sample mainly consisting of methylated fragments. Two different samples (*e.g*., case vs. control, tumor vs. normal, *etc*.) interrogated by the DMH protocol are then co-hybridized to generate a two-color image.

The literature is rich with discussion regarding varying experimental, hybridization, and technological effects that contribute non-biologically relevant signal (or noise) to the measured probe intensity. The fluorescent dyes employed in the sample labeling (most often Cy3 and Cy5) behave differently in a hybridization experiment (e.g., different incorporation rate and photo-bleaching rate) [10,11]. Biases that vary across or are correlated with position on the array are the most often cited array effects [10,12], and are attributed to the differences among print-tips on the array printer and the strike pattern over the course of the probe printing process. DNA fragments may bind to array probes with only partial complementarity. This cross-hybridization results in higher than expected probe signals [13,14].

Probe-target binding efficiencies associated with the probe sequence construct also contribute bias to array signals [15,16]. This is likely due to the higher energy needed to dissociate guanine (G) and cytosine (C) with three hydrogen bonds, as opposed to thymine (T) and adenine (A) with only two hydrogen bonds. A possible source of signal bias unique to the DMH protocol is associated with restriction cut-site density in the genomic neighborhood surrounding a probe's target region. It is reasonable to suspect that DMH samples may consist of a higher proportion of fragments with few restriction recognition sites between the PCR linkers since all restriction sites have to be methylated before the fragments can be amplified. It is potentially necessary to give more weight to probes with targets surrounded by many restriction sites. In this paper we develop a linear model that attempts to capture probe-sequence effects as well as dye-bias and restriction cut-site density effects in microarray studies obtained from DMH experiments. The microarrays used in these studies were printed using Agilent's SurePrint technology which utilizes the non-contact inkjet approach to generate probes, and thus spotting effects due to surface tension interactions and print-tip variability is a nonissue. Effects associated with cross-hybridization are best dealt with during the background correction of microarray preprocessing and our model assumes that this issue has been addressed.

There have been two well-accepted preprocessing strategies for gene expression and ChIP-chip microarray data that correct for probe-sequence effects: GC-RMA [15] and MAT [16], respectively. GC-RMA is a model-based background correction approach for Affymetrix gene expression arrays. The probe-target binding affinity *α *is modeled as a sum of position-dependent base effects:

(1) $$\alpha =\sum_{k=1}^{25}\sum_{j\in \{A,C,G,T\}}f_{5}(j,k)I(b_{k}=j),$$

where *k *indicates the position along the probe; *j *indexes the nucleotide base letter; *b*_*k *_represents the nucleotide base of the probe at position *k*; *I*(*b*_*k *_= *j*) is the indicator function that is 1 when the equality within the argument holds and is zero otherwise; and *f*_5_(*j*, *k*) captures the affinity of base *j *in position *k *and is fit to the data using a spline with 5 degrees of freedom.

MAT is a model-based analysis method for Affymetrix tiling-arrays hybridized with DNA samples from ChIP-chip studies. In the MAT model, the probe baseline intensity *m *is estimated via a linear combination of position-dependent base effects as well as target copy number:

(2) $$m=\alpha n_{T}+\sum_{k=1}^{25}\sum_{j\in \{A,C,G\}}\beta_{jk}I(b_{k}=j)+\sum_{j\in \{A,C,G,T\}}\gamma_{j}n_{j}^{2}+\delta \log(c)$$

where *k*, *j*, *b*_*k*_, and *I*(*b*_*k *_= *j*) are as in Equation 1; *n*_*j *_is the abundance of nucleotide *j *in the probe's sequence; *α *is the baseline value with respect to the amount of Ts in the sequence; *β*_*jk *_is the effect of each nucleotide *j *at each position *k*; *γ*_*j *_is the effect of the squared abundance of nucleotide *j*; and *δ *is the effect of the log of the probe copy number *c*.

In this work we propose two model-based approaches for signal correction of DMH data similar to those described above. We show that position-dependent base effects as well as dye-interaction and cut-site density effects are significant. The results are comparable between the two models; however, the interpretation of the parameters and subsequently their biological significance differ.

## Results

Two models are proposed which address the probe-sequence binding affinities in two different ways. The first, herein referred to as the *full-model*, is similar to the MAT model in that the effect of each nucleotide at each position is estimated. The second model, herein referred to as the *quadratic-model*, is similar to the GC-RMA model in that the nucleotide effect is modeled as a quadratic polynomial with respect to sequence position. For a more detailed description of either model, refer to the Methods section.

In order to assess the appropriateness of either model for DMH preprocessing, we fit the model to DMH microarray data obtained from the LBNL 51 Breast Cancer Cell Lines [17]. For readability we only discuss in detail the results from estimating parameters with respect to 9 of the LBNL-DMH data sets selected randomly.

### Nucleotide effect

#### Full-model

**Figure 1:**
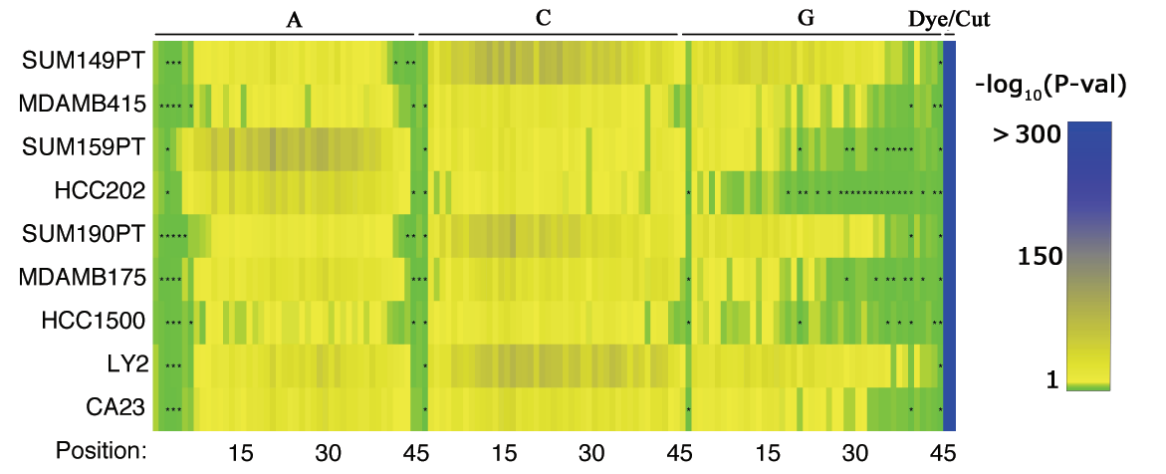
**PvalParameterEstimatesFullModelESS.eps**. Heat map depicting the statistical significance of the parameters for the full-model when fit to the LBNL-DM'9 data. The first 3 blocks, 45 columns each, represent the parameters associated with the effect of adenine (A), cytosine (C), and guanine (G), respectively, with distance along the sequence moving from left to right. The final 2 columns represent the parameters associated with the cut-site density and dye bias, respectively. The -log_10 _of the *p*-values for the estimates across the 9 samples are represented by a shade of green, yellow, or blue denoting *p*-values.

In the full-model there are a total of 138 parameters: 3 blocks of 45 parameters each are associated with the position within the probe sequence of nucleotides A, C, and G, respectively. The other three parameters associated with the dye, restriction cut-site density, and amount of nucleotide T. The majority of the parameters in the full-model are significantly different from zero (see Figure 1). Of exception are the parameters associated with the effects of the nucleotides at the 5' and 3' ends of the probe sequence. These parameters have relatively larger *p*-values and in many cases the effects are not significantly different from zero (*i.e*., *p*-val > 0.01 as denoted by black dots in Figure 1). This result supports the premise that binding events in the central portion of the probe are much stronger than events occurring at the tail end of the probe sequence and thus have a more significant effect on probe signal. The range of the nucleotide parameters across all experiments is -0.31 to 0.279, while the observed data ranges between 1 and 16 with the central 50% of the values ranging between 8.35 and 11.24 across all nine samples. The cofficient of variation of the the parameter estimates across the 9 samples was less than 0.5 in all but two of the parameters (which were in the 5' and 3' of the model). The cofficient of variation for the majority of the parameters was less than 0.15.

#### Quadratic-model

**Figure 2:**
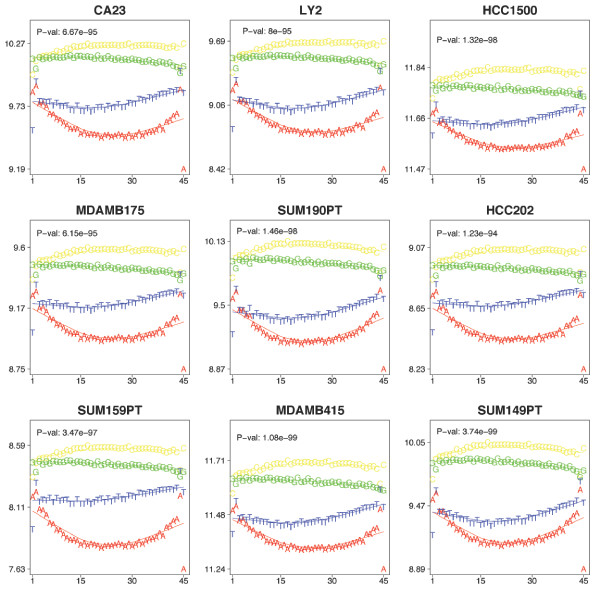
**SequenceRelationship2Intensity.eps**. The expected effect of A (red), C (yellow), G (green), and T (blue) at each 45-mer probe nucleotide position on probe intensity for the six samples. Plotted are the marginal average probe intensities (Cy5 channel only) with respect to probes with the same nucleotide at the given position. Printed *p*-values are associated to the ANOVA for the 4 different nucleotides.

**Figure 3:**
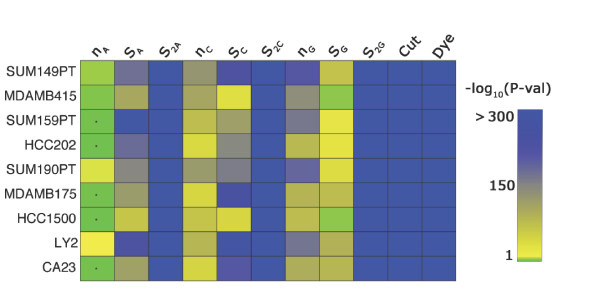
**PvalParameterEstimatesReducedModelESS.eps**. Heat map depicting the statistical significance of the parameters for the quadratic-model when fit to the LBNL-DMH'9 data. The first 3 blocks, 3 columns each, represent the parameters associated with coeficients of the quadratic fit to the effect of adenine (A), cytosine (C), and guanine (G), respectively, with respect to position in the probes sequence. The final 2 columns represent the parameters associated with the cut-site density and the dye bias, respectively. The -log_10 _of the *p*-values for the estimates across the 9 samples are represented by a shade of green, yellow, or blue denoting *p*-values.

The statistical insignificance of nucleotide effects near the ends of the probes and the apparent parabolic relationship between the expected probe intensity and the position of a given nucleotide within the probe sequence (see Figure 2) lead to the proposal of the quadratic-model. In the quadratic-model there are a total of 12 parameters, three of which are cofficients for each of the three quadratic relationships associated with nucleotide position within the probe sequence, giving rise to nine of the parameters. The remaining parameters were associated with the dye, restriction cut-site density, and abundance of thymine. Nearly all of the parameters of this model are significantly different from zero across the 9 samples (see Figure 3). The quadratic model was fit to standardized data; therefore, the estimated parameters are directly comparable. All of the effects, save *n*_*A*_, have relatively large absolute estimates (see Table 1). The estimated effects are fairly stable across the 9 samples with average variance well below the empirical probe variance across arrays. The cofficient of variation for all but one of the parameters was less than 0.5 (see Table 1). The majority of the parameters had cofficient of variation less than 0.35.

**Table 1:** Estimated cofficients for quadratic-model

Estimated Cofficients									
Cell Line	*n*_*A*_	*s*_*A*_	*s*_2*A*_	*n*_*C*_	*s*_*C*_	*s*_2*C*_	*n*_*G*_	*s*_*G*_	*s*_2*G*_

CA23	0.045	-1.051	0.618	0.352	1.505	-1.108	0.475	0.911	-0.824

LY2	0.11	-1.371	0.879	0.445	1.609	-1.14	0.58	0.87	-0.77

HCC1500	0.008	-0.983	0.707	0.526	0.836	-0.646	0.525	0.237	-0.271

MDAMB175	0.027	-0.908	0.54	0.291	1.363	-1.01	0.379	0.73	-0.693

SUM190PT	0.12	-1.29	0.914	0.617	1.417	-1.094	0.714	0.621	-0.652

HCC202	-0.027	-1.214	0.801	0.3	1.153	-0.883	0.398	0.461	-0.499

SUM159PT	-0.031	-1.712	1.222	0.425	1.054	-0.841	0.581	0.44	-0.516

MDAMB415	0.079	-1.158	0.813	0.617	0.678	-0.554	0.656	0.22	-0.294

SUM149PT	0.084	-1.206	0.908	0.544	1.448	-1.082	0.644	0.762	-0.721

Cofficient of Variation	1.216	0.197	0.241	0.276	0.26	0.23	0.211	0.442	0.344

Nucleotide effect cofficient estimates for the quadratic-model across the LBNL-DMH'9 data

To provide an interpretation of the observed effects, we can compute the expected baseline value of a probe with sequence comprised completely of one of the nucleotides A, C, or G. The predicted baseline signal of a hypothetical probe comprised of 100% adenine is half that of a hypothetical probe comprised of either 100% cytosine or guanine. This supports the biological premise that nucleotide effects can be explained by the extra hydrogen bond between cytosine and guanine.

### Restriction Density

In both models considered, the estimated restriction cut-density was statistically significant. The estimated values were relatively smaller, in absolute value, than the estimated nucleotide effects (-0.362 ± 0.068). The estimates are extremely robust across the nine samples with a standard deviation of 0.03. The estimated values are negative, corresponding with the hypothesis that loci with a larger density of cut-sites should have reduced expected signal intensity. With all other factors held, there is a greater than 1.5-fold predicted decrease in intensity for each increase of 10 cut-sites.

### Dye effect

In both the full- and quadratic-model the dye effect was significant (statistically and biologically). The estimated value of the dye effect is highly variable with an interquartile range 0.6.

### Comparison

Many methods have been proposed for the normalization of dual-channel microarray data, but typically these procedures neglect any effect related to probe sequence information. A commonly employed method is *M *– *A *loess normalization [18] that assumes that most probes should have similar value between the two channels. Other standardization procedures such as median adjustment and QQ-normalization [11] have also been employed to normalize multiple arrays before across-array comparisons are conducted. A more recent method, MA2C, has been proposed for the normalization of dual-channel arrays that takes into account the GC-content of the probes [19].

**Figure 4:**
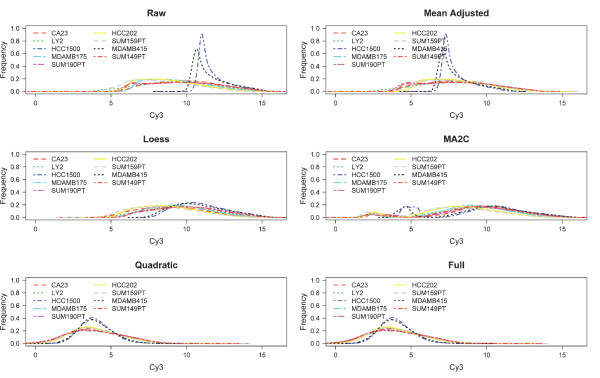
**Histograms.eps**. Distribution of Cy3 signal for the 9 arrays after using different signal correction methods: mean adjusted, loess, MA2C, the quadratic-model, and the full-model.

Figure 4 demonstrates that our proposed method standardizes the data much better than above proposed methods. On all 9 arrays considered, a pooled normal sample was hybridized on the Cy3 channel. Therefore, this channel should in theory be identical across the 9 arrays. Note that the raw signal from arrays hybridized with HCC1500 and MDAMB415 on the Cy5 channel have significantly different distributions of the Cy3 signal from the other arrays. This is likely due to technical issues related to the scanning of the arrays. Both the quadratic and full model standardization approaches perform the best at correcting the abnormal signal of these two arrays: all 9 arrays have the same mean and the variance of the outlier arrays is most similar to the other arrays.

**Figure 5:**
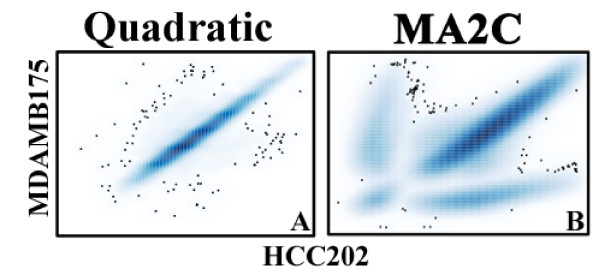
**MDAMBVSHCC.eps**. Cy3 signals, corrected via the quadratic-model (A) and MA2C (B), from the arrays hybridized with MDAMB175 and HCC202 are plotted against each-other. The Pearson's correlation for the quadratic-model data and the MA2C data are .98 and .49, respectively.

Figure 5 demonstrates the similarity of the Cy3 intensities between arrays after corrections according to the quadratic model, with a correlation cofficient of 0.98. Only the comparison of two arrays is shown; however, this plot is highly similar to the other pair-wise scatter plots (see Additional Files 1 and 2). On the other hand, correlation between arrays are actually reduced by the MA2X normalization procedure: by inspection of the scatter plots in figures 5 and Additional File 2, it appears that the majority of the probes are correlated across arrays; however, there are a significant subset of probes that have significant differences in signal intensity between arrays.

## Discussion and Conclusion

In this paper we have described two separate though related models for within-slide correction of signal effects associated with probe sequence construct. The first model assumes independence of positional effects, while the second model assumes a quadratic relationship in terms of nucleotide position. In either model, almost all parameters were significantly different from zero.

The two models correct for signal effects associated with probe sequence construct in an approach similar to that in the GC-RMA [15] and MAT [16] models developed for gene expression and ChIP-chip data, respectively. The results presented in either paper demonstrate that the probe sequence effect estimates are statistically significant; however, their estimated values are not biologically relevant. As the portion of predicted baseline signal associated with probe sequence in the GC-RMA model is relatively small, it contributes minimally to signal correction for gene expression data and could likely be ignored without detriment to the results purported in [15]. Similarly, the small sequence effects presented in [16] suggest that the overall baseline signal in ChIP-chip studies is explained by the other parameters in the MAT model, *i.e*, abundance of thymine, the squared abundance of each of the four nucleotides, and probe copy number.

**Figure 6:**
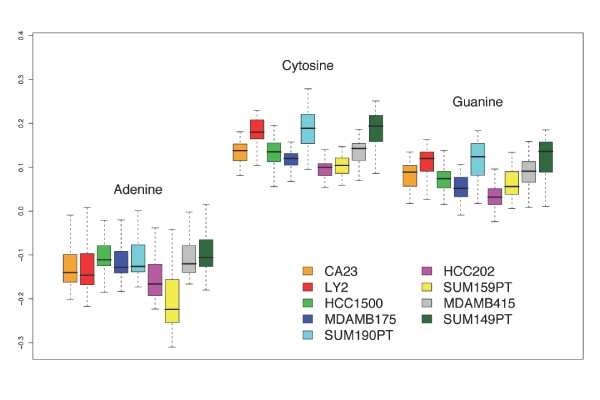
**BoxPlotCofficientsFullModel.eps**. Box-and-whisker plot for the estimated nucleotide effect across position. The range of values is considered separately for each of the 9 samples.

The extremely small *p*-values associated with the majority of the parameters of the full-model support their statistical significance as well as the appropriateness of the proposed model. However, the relatively small estimated values for these parameters (see Figure 6 and Additional File 3) are close to 0, and thus their biological significance are suspect. The individually estimated regression intercept was 6.818523 ± 0.54 while the estimates for the nucleotide effects were 0.05 ± 0.01, 0.03 ± 0.01, and 0.02 ± 0.01 for A, C, and G, respectively. Thus the nucleotide effects are statistically significant for the full-model but each individual effect contributes almost nothing to the overall expected baseline signal for a probe. This is due in large part to the unlikeliness that the the nucleotides contribute independently to the observed signal. In fact, the cumulative values in the full-model are biologically significant, that is, when the parameters are added in order to predict the baseline intensity for a given probe, the value is relatively large in comparison to the individual effects at each location.

Unlike the full-model, the estimated parameters of the quadratic-model are both statistically and biologically significant. In particular, when the model is fit to the standardized data, the degree-one and -two effects have estimated values near to 1 for many of the nucleotides across the 9 samples (see Table 1). Further, the quadratic-model is able to capture the cumulative effect of the nucleotides in a probe's sequence while also capturing the positional effect observed in Figure 2. Thus, we propose that the quadratic-model (as opposed to the full-model) more appropriately characterizes the nucleotide effect observed in DMH studies.

Interpretation of the models presented in this paper can provide some insight into some of the peculiarities of hybridization experiments. As is observable in Figure 2, the average signal for probes with adenine and thymine in the 3-prime and 5-prime ends, respectively, do not fit the general trend of the plot and are outliers. The effects of adenine are directly modeled in both models presented here. In the full model, the estimates of the adenine effect for the first 5 positions is relatively unstable across the 9 samples: 3 out of the 5 estimates have cofficient of variation greater than 0.6. A similar story unfolds in the quadratic-model in that the only parameter with a large cofficient of variation is that associated with the number of adenine nucleotides in the probe. These values suggest that there is a larger than expected variability in signal associated with probes with adenine nucleotides in their tails. This effect may be explained in part by the weaker binding between adenine and thymine; however, we suspect the effect is likely more complex. It has been suggested that the dye effect does not vary constantly across the range of signal intensity but is instead correlated with average signal intensity across the two colors [11]. As an alternative to the approach herein described for capturing the dye effect, this relationship may be modeled by a step function with respect to the observed probe intensity with steps at say the 1^*st*^, 2^*nd*^, and 3^*rd *^quartile. Such an approach is appealing, as it incorporates the previously observed relationship of dye effect and probe signal intensity. However, an interaction between dye and nucleotide composition is neglected. Though such an interaction is easy to describe mathematically and could be estimated from the data, the additional parameters would likely lead to over-fitting as was likely the issue with the full-model.

Another alternative to modeling dye-effect and nucleotide effect in concert would be to first correct for dye-effect in a non-parametric manner and then estimate the nucleotide effects using the dye-corrected data for the observed values. For example, one could employ the dye-correction strategy proposed in [10,11] in which the dye-effect is modeled by a *loess *curve in terms of average log_2 _probe intensity across the two channels. Care must be taken when correcting for dye effect in this manner, for in our experience, we have seen that this approach to dye-correction can introduce unexpected noise. For instance, correlations between a probe's spatial location on the array and the ranking of its N value have been observed (data not shown).

## Methods

### DMH

Differential methylation hybridization (DMH) [20] has been developed to determine the global methylation status of test and control genome. For a detailed description of the protocol used in the analyzed data, see [9]. Briefly, samples are sonicated in order to reduce genomic complexity. Fragments are end-repaired and linkers are ligated to the blunted fragments. Methylation-sensitive restriction enzymes *Hpa*II (CCGG) and *HinP*1I (GCGC) are used to cleave sonicated fragments containing unmethylated restriction sites. The enzyme-interrogated sample is amplified using PCR: because the PCR primers are designed against the ligated linkers, only uncleaved fragments will be amplified, producing amplicons enriched in methylated fragments. The amplicons are indirectly coupled with either Cy3 (G: green) or Cy5 (R: red) fluorescent dyes and the two labeled samples are co-hybridized onto the microarray.

### CGI-array

The Agilent 244K Human CpG Island Microarray (CGI-array) was employed for the high-throughput screening of aberrant methylation. The array tiles over 27,000 CGIs with 237,220 probes in or within 95 bp of a CpG Island. As opposed to the Affymetrix arrays, the probe lengths on the Aglilent CGI-array vary from 45 to 60 base pairs in length with the majority of probes (over 80%) 45 bp in length. Arrays were scanned using the Axon scanner with GenePix Pro 6.0 software.

### Cell lines

DMH analysis was performed on the LBNL 51 Breast Cancer Cell Lines [17]. These cell lines demonstrate a broad range of genomic, transcriptional, and biological heterogeneity and thus are useful models for investigating epigenetic characteristics in breast cancer. Of the 51 DMH data sets, 9 were used as the use-case data set (LBNL-DMH'9) for assessing the significance and appropriateness of the proposed modeling method. These 9 were chosen randomly from the initial population of 51 data sets.

### Preprocessing

Signal intensity for a given probe is due to fluorescent signals from labeled DNA probes (true complementary hybridization to the DNA targets) as well as various background signals. The scanning software provides an intensity value for background signal that is the summation of: 1) fluorescent intensities from the microarray substrate; 2) labeled DNA that cross-reacts with the substrate and not the considered probe target; 3) labeled DNA fragments that bleed over from neighboring probes; and 4) the occasional dye blob. This background signal is subtracted from the foreground signal for each probe. Occasionally a probe's foreground signal is less than the background signal or the probe is flagged for some other reason by the scanning software. In these situations, the missing probe signal is estimated to be the median signal value of the probes targeting a region 500 bp upstream and downstream of the given probe's DNA target.

### Full model

Motivated by the probe behavior model proposed by Johnson *et al *[16] for ChIP-chip data, we propose the following model that estimates the expected baseline signal from a DMH microarray experiment:

(3) $$p_{d}=\alpha_{0}+\sum_{j\in \{A,C,G\}}\left(\sum_{k=i}^{l}\beta_{jk}I(b_{k}=j)\right)+\gamma \chi +\delta I(d=G),$$

where

• *p*_*d *_is the expected baseline log transformed probe value for either the Cy3 (*d *= *G*) or the Cy5 (*d *= *R*) channel

• *k *indicates the position along the probe

• *l *denotes the probe length (45 ≤ *l *≤ 60)

• *j *indicates the nucleotide base letter

• *α*_0 _is the mean baseline signal across the array

• *I*(*b*_*k *_= *j*) and *I*(*d *= *G*) are indicator functions that are 1 when the equality in the argument holds and 0 otherwise

• *χ *is the number of methyl-sensitive restriction cut-sites located within a 1000 bp window centered at the probe

• *γ *is the effect of the cut-site frequency

• and *δ *is the global dye effect.

### Quadratic-model

Upon inspection of the *β*_*jk *_estimates of the nucleotide-position effect in the above model as well as the relationships evident in Figure 2, it was deemed appropriate to model the base-position effect as a quadratic polynomial. The use of a polynomial model is similar to that proposed in the GC-RMA approach described in [15], though the degrees differ as well as interpretation. Formally, the model for predicting a fixed probe's baseline signal is given by:

(4) $$\begin{array}{c} p_{d}=\alpha_{0}n_{T}+\frac{1}{l}\sum_{j\in \{A,C,G\}}\sum_{k=1}^{l}\left(\beta_{j0}+\beta_{j1}k+\beta_{j2}k^{2})I(b_{k}=j\right)+\gamma \chi +\delta I(d=G)\\ =\alpha_{0}n_{T}+\sum_{j\in \{A,C,G\}}\left(\beta_{j0}n_{j}+\beta_{j1}S_{j}+\beta_{j2}S_{2j}\right)+\gamma \chi +\delta I(d=G), \end{array}$$

where

• *p*_*d*_, *j*, *k*, *l*, *I*(*b*_*k *_= *j*), *I*(*d *= *G*), *δ*, *χ *and *γ *are the same as in Equation (3)

• *n*_*T *_denotes the number of thymine nucleotides in the probes sequence

• *β*_*ji*_, *i *∈ {0, 1, 2}, are the cofficients for the polynomial contribution of base *j *at position *k*

• *n*_*j *_is the abundance of nucleotide *j *in the probe sequence divided by *l*

• and *S*_*j*_, and *S*_2*j*_, is the sum of the position, and the sum of the square of the position, of base *j *within the sequence of the probe divided by *l*, respectively.

Unlike the full-model, the independent variables in the quadratic-model take on values other than 0 and 1. To allow for interpretation of the results, the model is fit using explanatory variables that are standardized, *i.e*.,

(5) $$p_{d}=\alpha_{0}{n^{\prime }}_{T}+\sum_{j\in \{A,C,G\}}\left(\beta_{j0}{n^{\prime }}_{j}+\beta_{j1}{S^{\prime }}_{j}+\beta_{j2}{S^{\prime }}_{2j}\right)+\gamma \chi +\delta I(d=G),$$

where ${n^{\prime }}_{j}$, ${S^{\prime }}_{j}$, and ${S^{\prime }}_{2}j$ are the standardized form of *n*_*j*_, *S*_*j*_, and *S*_2_*j*, respectively, so as to have mean 0 and variance 1.

### Model fitting

Estimation of probe behavior takes advantage of the expectation that the majority of probes will not target DNA regions that survive the methylation interrogation by the restriction enzymes. Thus, the majority of the observed signal is due to the varying biases in the experiment or hybridization, *i.e*., the exact features being captured by the two models. Further, there are nearly a half-million observations for a given microarray, allowing for a robust and accurate estimation of the different effects in the model. Model fitting is performed on each array separately via linear least squares.

### Estimates of parameter significance

Assuming that the observed errors are normally distributed, the parameter estimates will belong to a *t*-distribution. As there are well over 200 K degrees of freedom in either model proposed, the *t*-distribution is well approximated by a normal distribution; therefore, all *p*-values are estimated using a normal distribution. For the *j*^*th *^parameter *ρ*_*j *_in either model, the variance *σ*_*j *_is estimated by ${\hat{\sigma }}_{j}^{2}=\frac{RSS}{m-n}{\left(X^{T}X\right)}_{ij}^{-1}$, where *RSS *is the regression sum of squares (also known as the sum of squared residuals). Therefore, *ρ*_*j*_/${\hat{\sigma }}_{j}$ follows a standard normal distribution.

## Authors' contributions

DP developed and implemented the models, performed all statistical analyses, and drafted the manuscript. PY was involved in the data collection and helped in the preparation of the manuscript. SL provided advice on the project, revised the draft manuscript, and lead the project. THMH oversaw the project and revised the draft manuscript. All authors read and approved the final document.

## Supplementary Material

Additional file 1 **In the top right corner of the plotted matrix, the Cy3 signals corrected with respect to the quadratic-model are plotted against each-other.** The Pearson's correlation for each of the 36 comparisons is denoted in the plots reflection across the diagonal. The samples compared in each of the plots are denoted along the diagonal of the matrix. Click here for file

Additional file 2 **In the top right corner of the plotted matrix, the Cy3 signals corrected via MA2C are plotted against each-other.** The Pearson's correlation for each of the 36 comparisons is denoted in the plots reflection across the diagonal. The samples compared in each of the plots are denoted along the diagonal of the matrix. Click here for file

Additional file 3 **Estimated cofficients for full-model**. As there are 138 parameters in the full model, the table of their estimates is much to large to print to a standard page. This table can be found in the pdf file FullModelTable.pdf. The LATEX file that generated the pdf is FullModelTable.tex. Individual nucleotide cofficient estimates for each of the three nucleotides adenine, cytosine, and guanine in the full-model across the LBNL-DMH'9 data. Click here for file
