# Supplementary material for: Probe signal correction for differential methylation hybridization experiments
Source: BMC Bioinformatics. 2008 Oct 23;9:453. doi: 10.1186/1471-2105-9-453 (PMC2603337; doi:10.1186/1471-2105-9-453)
Supplement: Additional file 3 — Estimated cofficients for full-model. As there are 138 parameters in the full model, the table of their estimates is much to large to print to a standard page. This table can be found in the pdf file FullModelTable.pdf. The LATEX file that generated the pdf is FullModelTable.tex. Individual nucleotide cofficient estimates for each of the three nucleotides adenine, cytosine, and guanine in the full-model across the LBNL-DMH'9 data. [file 1471-2105-9-453-S3.pdf]

Estimated Coefficients

Adenine

Position	1	2	3	4	5	6	7	8	9	10	11	12	13	14	15	16	17	18	19	20	21	22
CA23	0.102	-0.037	-0.009	-0.01	-0.027	-0.071	-0.058	-0.083	-0.115	-0.107	-0.117	-0.112	-0.126	-0.152	-0.129	-0.108	-0.148	-0.148	-0.154	-0.162	-0.194	-0.165
LY2	0.099	-0.031	0.008	-0.023	-0.032	-0.055	-0.043	-0.095	-0.093	-0.097	-0.134	-0.124	-0.122	-0.154	-0.136	-0.123	-0.155	-0.13	-0.157	-0.162	-0.204	-0.149
HCC1500	0.089	-0.042	-0.021	-0.03	-0.034	-0.079	-0.04	-0.1	-0.074	-0.117	-0.141	-0.134	-0.095	-0.113	-0.101	-0.1	-0.142	-0.111	-0.118	-0.136	-0.176	-0.125
MDAMB175	0.073	-0.015	-0.004	-0.021	-0.02	-0.069	-0.049	-0.095	-0.095	-0.092	-0.13	-0.101	-0.104	-0.135	-0.101	-0.11	-0.138	-0.124	-0.141	-0.135	-0.167	-0.145
SUM190PT	0.106	-0.027	-0.002	0.001	-0.004	-0.015	-0.074	-0.077	-0.084	-0.092	-0.135	-0.126	-0.127	-0.134	-0.107	-0.121	-0.12	-0.134	-0.143	-0.14	-0.171	-0.133
HCC202	0.101	-0.042	-0.007	-0.04	-0.039	-0.117	-0.093	-0.121	-0.138	-0.15	-0.2	-0.158	-0.155	-0.207	-0.164	-0.17	-0.189	-0.164	-0.189	-0.2	-0.223	-0.196
SUM159PT	0.094	-0.041	-0.003	-0.056	-0.075	-0.139	-0.123	-0.183	-0.193	-0.204	-0.241	-0.215	-0.215	-0.269	-0.208	-0.225	-0.254	-0.235	-0.263	-0.255	-0.31	-0.258
MDAMB415	0.094	-0.023	-0.002	-0.036	-0.007	-0.062	-0.03	-0.097	-0.091	-0.074	-0.127	-0.136	-0.098	-0.141	-0.118	-0.079	-0.154	-0.123	-0.14	-0.126	-0.164	-0.133
SUM149PT	0.094	-0.028	0.016	-0.018	-0.007	-0.065	-0.054	-0.101	-0.103	-0.099	-0.115	-0.106	-0.107	-0.141	-0.121	-0.115	-0.132	-0.132	-0.117	-0.135	-0.18	-0.126
Coefficient of Variation	0.102	0.298	3.888	0.654	0.811	0.478	0.470	0.297	0.332	0.345	0.287	0.258	0.295	0.299	0.264	0.342	0.254	0.257	0.284	0.259	0.231	0.273

Position	23	24	25	26	27	28	29	30	31	32	33	34	35	36	37	38	39	40	41	42	43	44	45
CA23	-0.154	-0.131	-0.14	-0.176	-0.14	-0.17	-0.181	-0.201	-0.16	-0.169	-0.183	-0.163	-0.16	-0.141	-0.146	-0.162	-0.164	-0.128	-0.143	-0.099	-0.085	-0.071	-0.076
LY2	-0.17	-0.179	-0.168	-0.186	-0.164	-0.181	-0.217	-0.205	-0.17	-0.204	-0.181	-0.165	-0.16	-0.154	-0.139	-0.175	-0.151	-0.146	-0.128	-0.117	-0.105	-0.071	-0.0556
HCC1500	-0.11	-0.119	-0.113	-0.118	-0.116	-0.136	-0.13	-0.185	-0.111	-0.136	-0.112	-0.119	-0.107	-0.129	-0.104	-0.128	-0.12	-0.106	-0.085	-0.072	-0.068	-0.067	-0.0346
MDAMB175	-0.136	-0.137	-0.128	-0.162	-0.135	-0.146	-0.156	-0.183	-0.141	-0.154	-0.15	-0.149	-0.143	-0.121	-0.136	-0.131	-0.13	-0.107	-0.096	-0.088	-0.077	-0.047	-0.0256
SUM190PT	-0.15	-0.14	-0.131	-0.141	-0.122	-0.135	-0.173	-0.159	-0.13	-0.153	-0.147	-0.138	-0.13	-0.147	-0.116	-0.127	-0.109	-0.107	-0.094	-0.071	-0.051	-0.029	-0.0056
HCC202	-0.203	-0.178	-0.186	-0.214	-0.166	-0.187	-0.198	-0.202	-0.19	-0.204	-0.198	-0.193	-0.178	-0.167	-0.175	-0.166	-0.16	-0.135	-0.137	-0.116	-0.083	-0.066	-0.0386
SUM159PT	-0.269	-0.254	-0.244	-0.271	-0.241	-0.266	-0.278	-0.283	-0.242	-0.253	-0.241	-0.242	-0.243	-0.224	-0.203	-0.206	-0.201	-0.162	-0.156	-0.113	-0.112	-0.076	-0.0556
MDAMB415	-0.114	-0.127	-0.148	-0.155	-0.089	-0.138	-0.144	-0.166	-0.128	-0.14	-0.122	-0.156	-0.119	-0.144	-0.104	-0.157	-0.137	-0.12	-0.101	-0.088	-0.064	-0.053	-0.036
SUM149PT	-0.127	-0.117	-0.11	-0.149	-0.104	-0.121	-0.145	-0.15	-0.121	-0.161	-0.106	-0.133	-0.099	-0.102	-0.095	-0.108	-0.105	-0.083	-0.06	-0.034	-0.039	-0.01	-0.0036
Coefficient of Variation	0.316	0.287	0.279	0.260	0.318	0.270	0.255	0.203	0.267	0.218	0.279	0.227	0.296	0.233	0.264	0.200	0.216	0.197	0.283	0.304	0.311	0.410	0.641

Cytosine

Position	1	2	3	4	5	6	7	8	9	10	11	12	13	14	15	16	17	18	19	20	21	22
CA23	0.081	-0.003	0.115	0.092	0.112	0.098	0.138	0.137	0.1	0.129	0.143	0.152	0.157	0.131	0.17	0.177	0.181	0.135	0.156	0.147	0.129	0.156
LY2	0.066	0.022	0.139	0.104	0.124	0.128	0.181	0.166	0.162	0.172	0.186	0.201	0.206	0.176	0.221	0.208	0.23	0.213	0.212	0.215	0.178	0.227
HCC1500	0.086	0.032	0.145	0.101	0.127	0.11	0.171	0.114	0.135	0.133	0.126	0.15	0.187	0.133	0.172	0.196	0.165	0.161	0.135	0.137	0.112	0.176
MDAMB175	0.029	0.004	0.105	0.056	0.112	0.073	0.12	0.118	0.092	0.121	0.13	0.142	0.147	0.113	0.157	0.142	0.158	0.12	0.134	0.124	0.1	0.131
SUM190PT	0.095	0.029	0.141	0.119	0.191	0.136	0.213	0.197	0.184	0.223	0.221	0.223	0.248	0.223	0.246	0.245	0.279	0.214	0.214	0.219	0.189	0.241
HCC202	0.054	-0.006	0.1	0.055	0.112	0.062	0.108	0.096	0.082	0.092	0.08	0.106	0.124	0.089	0.135	0.127	0.14	0.102	0.105	0.101	0.08	0.102
SUM159PT	0.067	0.006	0.122	0.073	0.109	0.088	0.136	0.112	0.088	0.113	0.124	0.135	0.137	0.098	0.147	0.123	0.143	0.103	0.122	0.107	0.086	0.108
MDAMB415	0.096	0.038	0.17	0.106	0.145	0.113	0.18	0.15	0.138	0.158	0.159	0.146	0.167	0.131	0.171	0.187	0.148	0.136	0.145	0.151	0.134	0.167
SUM149PT	0.068	0.036	0.158	0.105	0.161	0.157	0.194	0.182	0.179	0.197	0.218	0.221	0.245	0.208	0.243	0.212	0.251	0.215	0.231	0.231	0.177	0.24
Coefficient of Variation	0.068	0.036	0.158	0.105	0.161	0.157	0.194	0.182	0.179	0.197	0.218	0.221	0.245	0.208	0.243	0.212	0.251	0.215	0.231	0.231	0.177	0.24

Position	23	24	25	26	27	28	29	30	31	32	33	34	35	36	37	38	39	40	41	42	43	44	45
CA23	0.15	0.162	0.156	0.138	0.16	0.149	0.131	0.114	0.162	0.142	0.136	0.157	0.117	0.142	0.13	0.146	0.112	0.1	0.139	0.139	0.153	0.097	0.1
LY2	0.211	0.206	0.21	0.175	0.204	0.211	0.17	0.165	0.211	0.179	0.191	0.215	0.2	0.186	0.147	0.18	0.167	0.122	0.189	0.178	0.171	0.147	0.12
HCC1500	0.167	0.148	0.148	0.141	0.16	0.175	0.124	0.097	0.141	0.135	0.164	0.155	0.136	0.113	0.14	0.111	0.108	0.072	0.117	0.128	0.134	0.077	0.056
MDAMB175	0.136	0.124	0.132	0.097	0.13	0.126	0.115	0.077	0.125	0.111	0.117	0.124	0.117	0.108	0.108	0.133	0.102	0.068	0.133	0.128	0.136	0.123	0.103
SUM190PT	0.22	0.197	0.223	0.198	0.223	0.221	0.181	0.17	0.2	0.175	0.174	0.189	0.176	0.148	0.163	0.172	0.136	0.101	0.158	0.152	0.154	0.15	0.103
HCC202	0.105	0.106	0.123	0.074	0.109	0.109	0.087	0.065	0.09	0.091	0.084	0.108	0.102	0.074	0.081	0.1	0.069	0.062	0.111	0.105	0.116	0.113	0.084
SUM159PT	0.104	0.103	0.122	0.079	0.121	0.107	0.08	0.063	0.099	0.091	0.098	0.107	0.086	0.079	0.099	0.091	0.081	0.059	0.11	0.125	0.109	0.111	0.077
MDAMB415	0.161	0.153	0.143	0.124	0.166	0.164	0.124	0.119	0.154	0.125	0.148	0.143	0.139	0.101	0.116	0.108	0.097	0.069	0.116	0.118	0.151	0.091	0.057
SUM149PT	0.226	0.221	0.247	0.201	0.215	0.219	0.194	0.175	0.212	0.189	0.201	0.183	0.195	0.159	0.178	0.16	0.146	0.108	0.159	0.163	0.139	0.135	0.086
Coefficient of Variation	0.226	0.221	0.247	0.201	0.215	0.219	0.194	0.175	0.212	0.189	0.201	0.183	0.195	0.159	0.178	0.16	0.146	0.108	0.159	0.163	0.139	0.135	0.086

Guanine

Position	1	2	3	4	5	6	7	8	9	10	11	12	13	14	15	16	17	18	19	20	21	22
CA23	0.119	0.026	0.109	0.098	0.104	0.102	0.125	0.107	0.091	0.102	0.113	0.131	0.111	0.083	0.128	0.135	0.121	0.08	0.096	0.098	0.065	0.086
LY2	0.12	0.054	0.139	0.119	0.12	0.114	0.152	0.132	0.128	0.124	0.135	0.164	0.144	0.129	0.162	0.152	0.157	0.151	0.129	0.15	0.098	0.147
HCC1500	0.106	-0.006	0.104	0.095	0.087	0.103	0.138	0.068	0.088	0.077	0.082	0.086	0.111	0.077	0.105	0.105	0.094	0.077	0.061	0.076	0.026	0.06
MDAMB175	0.077	0.031	0.081	0.06	0.095	0.067	0.091	0.081	0.055	0.072	0.078	0.107	0.091	0.06	0.092	0.083	0.086	0.061	0.052	0.07	0.034	0.067
SUM190PT	0.148	0.05	0.126	0.141	0.173	0.119	0.17	0.162	0.135	0.157	0.158	0.171	0.162	0.16	0.183	0.152	0.173	0.136	0.121	0.16	0.094	0.154
HCC202	0.113	0.014	0.083	0.06	0.096	0.047	0.078	0.055	0.051	0.043	0.047	0.071	0.051	0.04	0.066	0.071	0.056	0.043	0.021	0.043	0.019	0.03
SUM159PT	0.134	0.043	0.127	0.084	0.125	0.098	0.128	0.097	0.092	0.089	0.09	0.113	0.108	0.06	0.107	0.093	0.074	0.064	0.056	0.074	0.021	0.051
MDAMB415	0.115	0.046	0.137	0.088	0.13	0.128	0.159	0.13	0.12	0.12	0.113	0.106	0.132	0.084	0.11	0.141	0.091	0.091	0.079	0.106	0.066	0.095
SUM149PT	0.115	0.055	0.136	0.116	0.152	0.137	0.172	0.139	0.135	0.158	0.177	0.174	0.163	0.159	0.185	0.161	0.162	0.15	0.151	0.175	0.111	0.157
Coefficient of Variation	0.115	0.055	0.136	0.116	0.152	0.137	0.172	0.139	0.135	0.158	0.177	0.174	0.163	0.159	0.185	0.161	0.162	0.15	0.151	0.175	0.111	0.157

Position	23	24	25	26	27	28	29	30	31	32	33	34	35	36	37	38	39	40	41	42	43	44	45
CA23	0.112	0.104	0.096	0.074	0.09	0.085	0.066	0.068	0.101	0.089	0.058	0.057	0.047	0.05	0.057	0.051	0.051	0.027	0.053	0.043	0.051	0.043	0.017
LY2	0.148	0.126	0.126	0.111	0.124	0.12	0.084	0.108	0.122	0.11	0.099	0.105	0.111	0.091	0.055	0.073	0.073	0.042	0.09	0.061	0.063	0.059	0.026
HCC1500	0.09	0.094	0.073	0.053	0.075	0.09	0.06	0.046	0.072	0.091	0.065	0.074	0.053	0.023	0.058	0.028	0.048	0.017	0.044	0.048	0.07	0.032	0.015
MDAMB175	0.077	0.058	0.063	0.031	0.043	0.043	0.03	0.031	0.048	0.05	0.035	0.023	0.035	0.017	0.005	0.035	0.017	-0.009	0.04	0.019	0.036	0.033	0.006
SUM190PT	0.144	0.124	0.127	0.126	0.122	0.129	0.082	0.106	0.114	0.106	0.089	0.082	0.083	0.048	0.066	0.059	0.061	0.023	0.07	0.045	0.06	0.058	0.017
HCC202	0.048	0.031	0.042	0.01	0.04	0.026	0.002	0.018	0.017	0.025	0.007	0.007	0.025	-0.005	-0.005	0.012	-0.003	-0.024	0.032	0.015	0.033	0.028	0.003
SUM159PT	0.067	0.046	0.067	0.05	0.057	0.056	0.027	0.028	0.059	0.056	0.035	0.032	0.042	0.01	0.024	0.025	0.02	0.011	0.048	0.049	0.039	0.044	0.006
MDAMB415	0.128	0.113	0.078	0.071	0.113	0.098	0.065	0.08	0.104	0.091	0.069	0.054	0.072	0.044	0.053	0.04	0.047	0.034	0.069	0.06	0.072	0.035	0.008
SUM149PT	0.173	0.14	0.161	0.136	0.144	0.143	0.111	0.113	0.139	0.121	0.115	0.089	0.1	0.061	0.071	0.069	0.051	0.039	0.082	0.055	0.054	0.062	0.011
Coefficient of Variation	0.173	0.14	0.161	0.136	0.144	0.143	0.111	0.113	0.139	0.121	0.115	0.089	0.1	0.061	0.071	0.069	0.051	0.039	0.082	0.055	0.054	0.062	0.011
